# Distinct clinical phenotypes in a family with a novel truncating MEN1 frameshift mutation

**DOI:** 10.1186/s12902-022-00978-9

**Published:** 2022-03-14

**Authors:** Christoph Welsch, Anna Katharina Flügel, Susanne Rondot, Egbert Schulze, Ishani Sircar, Judith Nußbaumer, Jörg Bojunga

**Affiliations:** 1 Department of Internal Medicine 1, Goethe-University Hospital Frankfurt, Frankfurt am Main, Germany; 2 MVZ Labor Dr. Limbach & Kollegen GbR, Molecular Endocrinology, Heidelberg, Germany; 3 endokrinologikum Frankfurt, Frankfurt am Main, Germany

**Keywords:** Case report, MEN1, Truncating mutation, Frameshift, Clinical phenotype

## Abstract

**Background:**

MEN1 mutations can inactivate or disrupt menin function and are leading to multiple endocrine neoplasia type 1, a rare heritable tumor syndrome.

**Case presentation:**

We report on a MEN1 family with a novel heterozygous germline mutation, c.674delG; p.Gly225Aspfs*56 in exon 4 of the MEN1 gene. Diagnosis and clinical phenotyping of MEN1 was established by laboratory tests, ultrasound, biopsy, MRI imaging and endosonography. The clinical course of the disease was followed in the index patient and her family members for eight years. The mutation was associated with distinct clinical phenotypes in the index patient and three family members harboring p.Gly225Aspfs*56. Family members affected showed primary hyperparathyroidism but variable patterns of associated endocrine tumors, adrenal cortical adenomas, prolactinoma, multifocal pancreatic neuroendocrine tumors, insulinoma and nonsecretory neuroendocrine tumors of the pancreas. The mutation c.674delG; p.Gly225Aspfs*56 leads to a frameshift from codon 225 with early truncation of the menin protein. In silico analysis predicts loss of multiple protein-menin interactions in p.Gly225Aspfs*56, potentially rendering menin insufficient to control cell division and replication. However, no aggressive neuroendocrine tumors were observed in the follow-up of this family.

**Conclusions:**

We report a novel heterozygous MEN1 frameshift mutation, potentially causing (at least partial) inactivation of menin tumor suppression potential but lacking a genotype–phenotype correlation. Our study highlights the importance of personalized care with appropriate testing and counseling in MEN1 families.

## Background

Multiple endocrine neoplasia type 1 (MEN1) is a hereditary tumor syndrome characterized by tumorigenesis in multiple endocrine organs. MEN1 has an estimated incidence of 0.25% in random postmortem studies [1]. Inheritance of classic MEN1 follows an autosomal dominant pattern with a high degree of penetrance, with clinical and biochemical manifestations occurring in 80% of MEN1 patients and in more than 98% by the fifth decade of life [1]. The clinical criterion for the diagnosis of MEN1 is the presence of two of the three major neuroendocrine tumor (NET) types in an index patient, i.e., tumors of the parathyroid, pituitary, and/or pancreatic islet cells, or a MEN1-associated tumor in a family member of a patient with MEN1 [1]. Multiple parathyroid tumors with hyperparathyroidism are the most common manifestation with almost 100% penetrance at age 40 to 50 years, followed by functional or nonfunctional pancreatic neuroendocrine tumors in 85% of patients at age 50 years. In addition, patients potentially develop duodenal gastrinomas, thymic or bronchial carcinoid tumors, enterochromaffin cell-like gastric tumors, adrenocortical adenomas, lipomas, angiofibromas, angiomyolipomas as well as spinal cord ependymomas [2]. The MEN1 gene is located on the long arm of chromosome 11 (11q13). The gene consists of 10 exons and encodes a 610 amino acid protein termed menin [3, 4]. The MEN1 germline mutations reported to date are scattered throughout the 1830-bp coding region and can attenuate or abolish menin function [5, 6]. Menin is a tumor suppressor involved in various cellular processes and signaling cascades. As a scaffold protein, it coordinates the function of multiple proteins by direct and indirect interaction. Among those protein interaction partners are transcription activators and repressors, signaling proteins and proteins involved in cell cycle or DNA repair [7]. Huang and co-workers reported a crystal structure showing tethering of menin and the mixed lineage leukaemia protein 1 (MLL1), an oncogenic cofactor in gene transcription and leukaemogenesis, to chromatin binding factor lens epithelium-derived growth factor (LEDGF) [8]. The complex network of protein-menin interactions is affected by MEN1 missense mutations with pleiotropic effects that can impact tumor suppressor activity [7–9]. Here, we report a novel MEN1 germline mutation with a reading frame shift and premature truncation of the menin protein, potentially affecting protein interactions and causing multiple endocrine tumor patterns. We could not detect any genotype–phenotype correlation and no aggressive neuroendocrine tumor during several years of follow-up.

## Patients and methods

At the time of first presentation, the index patient is a 28-year-old woman, diagnosed with a prolactinoma 10 years ago, who consulted us for hypoglycemia. Diagnosis of insulinoma was established by fasting test and imaging of the pancreas. We did screening for hyperparathyroidism including assessment of plasma calcium and parathormone (PTH) concentrations as well as ultrasound imaging of the parathyroid glands. We obtained informed consent for family screening with genotyping and mutation analyses from the index patient and four family members (the father, brother, sister and the paternal aunt). The clinical course of disease was followed over 8 years in the index patient and her family members.

Molecular analysis was performed at the laboratory for human genetics (Heidelberg, Germany). Genomic DNA was prepared from peripheral blood leukocytes, amplified by PCR and sequenced by ABI 3031-xl DNA sequencer and Sanger sequencing. Multiplex ligation-dependent probe amplification analysis using the MLPA-Kit (MRC-Holland, Kit P 017-C1) was applied to detect deletions or duplications in MEN1 exon 1–10. Next generation sequencing (NGS) was performed using the MiSeq platform (Illumina Inc., San Diego, CA, USA). Analysis of exon 2–10 and exon/intron transitions of the MEN1 gene (MEN1 reference sequence: ENST00000315422) was amplified using multiplex-PCR. Bioinformatics sequence analysis was performed with NextSeq and SeqPilot software (JSI Inc.). PyMOL Molecular Graphics System (DeLano Scientific, San Carlos, CA, USA) was used for in silico 3D structure analysis and visualization.

## Case presentation

**Fig. 1:**
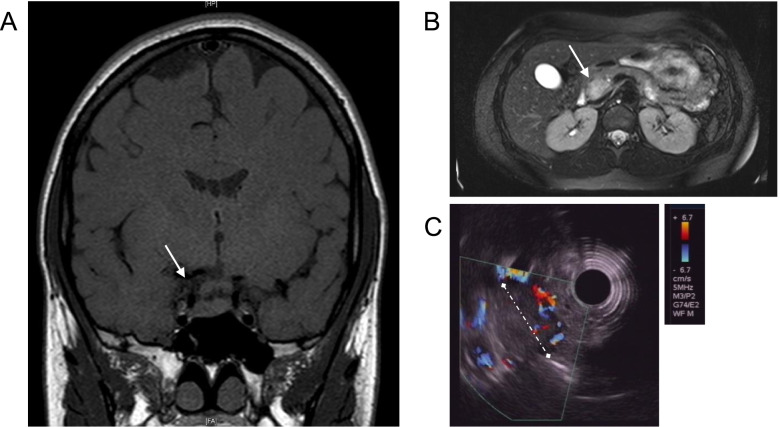
Imaging in the 28-year-old index patient. Imaging is showing an adenoma of the pituitary gland and insulinoma of the pancreas. **A** Magnetic resonance imaging (MRI) of the pituitary gland with tissue in lateral contact with the cavernous sinus; diameter of 5.0 × 7.5 × 4.2 mm. **B** T2-weighted MRI image of the abdomen documenting an inhomogeneous, hypervascularized, contrast-enhanced mass in the pancreatic head measuring 2.0 × 3.0 × 2.8 cm, consistent with an islet cell tumor. **C** Endoscopic ultrasound (EUS) showing color flow Doppler image of the corresponding hypervascularized lesion in the pancreatic head

**Fig. 2:**
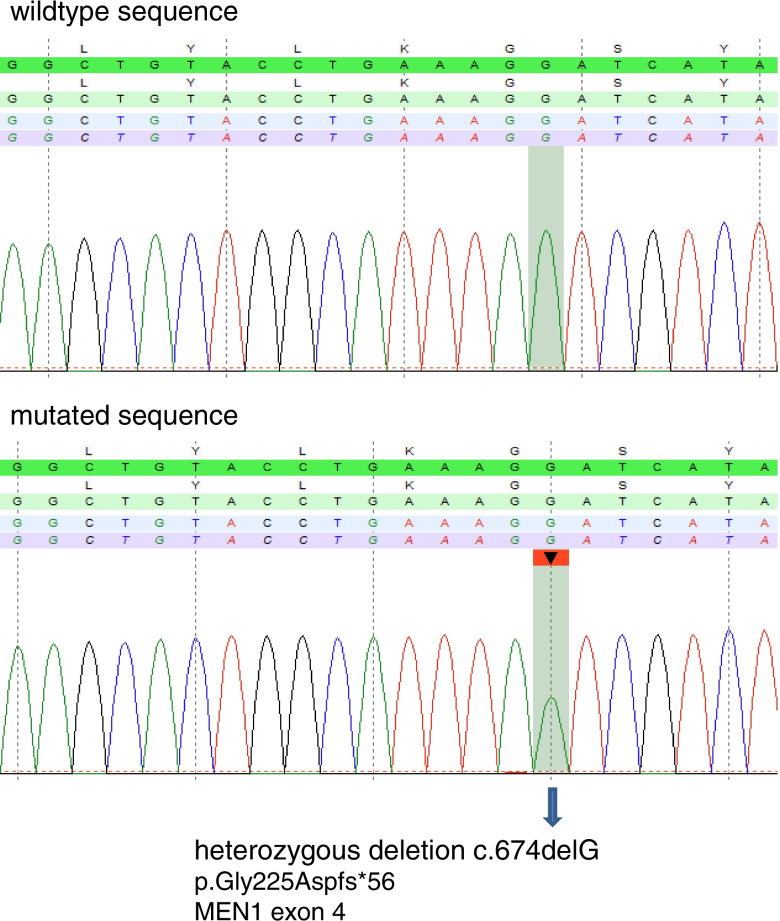
Sequencing of the MEN1 gene. Sequence analysis of exon 4 of the *MEN1* gene showing the presence of the heterozygous deletion c.674delG in the index patient (mutated sequence). The deletion is indicated by a decreased intensity of the deleted base (decrease in peak height). The c.674 position is indicated by green squares (the percentage near 50% indicates a heterozygous deletion of one nucleotide in the germline sequence). A gap starting at the deletion is visible in the sequence alignment of the different reads by SeqPilot and indicates a frameshift (not shown)

The index patient presented to our clinic for further evaluation after a temporary loss of consciousness due to recurrent hypoglycemia. Magnetic resonance imaging (MRI) showed the known prolactinoma of the pituitary with unchanged size (Fig. 1A). Fasting test revealed hypoglycemia with a blood glucose level of 2.7 mmol/l and insulin and C-peptide levels not suppressed (27.0 µIU/ml and 3.3 ng/ml, respectively) (Table 1). MRI of the abdomen and endoscopic ultrasound (EUS) (Fig. 1B and 1C) showed one lesion of about 3 cm in diameter in the head of the pancreas and another lesion of about 2.8 cm in diameter in the pancreatic tail. Serum markers for endocrine tumors, Gastrin and Chromogranin A, were within the normal range. Primary hyperparathyroidism was identified by laboratory testing and confirmed as multiglandular hyperparathyroidism. The patient underwent duodenopancreatic surgery with pylorus-preserving pancreaticoduodenectomy (PPPD) and removal of the pancreatic tail. Histology confirmed an insulinoma in the pancreatic head and a multifocal neuroendocrine tumor in the pancreatic tail, but no malignant neuroendocrine neoplasia of the pancreas. Sequence analysis of the MEN1 gene revealed a previously undescribed heterozygous point mutation in exon 4, c.674delG; p.Gly225Aspfs*56 (Fig. 2). The mutation causes deletion of the second nucleotide position of codon 225 and a resulting 55 amino acid frameshift (p.Gly225Aspfs*56). The consecutive amino acid sequence change leads to an early termination of translation and a resulting truncation of the menin protein at residue 280. Absence of this novel germline mutation was confirmed in a total of more than 100 healthy controls (own control database). We detected no other mutations in the MEN1 gene of the index patient.

**Table 1:** Baseline laboratory tests to confirm MEN1 in the index patient and family members

**Laboratory tests** (normal range)	**Index patient**	**Father**	**Brother**	**Sister**
Calcium mmol/l (2.09 – 2.54)	2.8	3.2	2.9	2.4
Phosphate mmol/l (2.6 – 4.5)	2.2	2.2	1.6	2.4
Albumine g/dl (3.5 – 5.2)	4.8	4.4	4.8	4.3
PTH pg/ml (15 – 65)	117.0	258.0	89.0	76.0
Gastrin pg/ml (13 – 115)	59.0	62.0	10.0	18.0
Chromogranin A U/l (< 35)	12.2	19.0	11.0	14.0
Prolactin mg/dl (4.6 – 21.4)	93.0	12.0	18.5	50.4
Fasting blood glucose mmol/l (82 – 115)	2.7	n/d	n/d	n/d
Insulin µIU/ml (2.6 – 24.9)	27.0	57.0	10.0	n/d
C-peptide ng/ml (1.1 – 4.4)	3.3	7.7	n/d	n/d
IGF-1 ng/ml (78 – 220)	206.0	167.0	179.0	135
25-OH Vitamin D3 ng/ml (10 – 25)	13.0	4,1	14.0	< 4.0

*IGF*-1 insulin-like growth factor; *PTH* parathormone; *n/a* not available; *n/d* not determined

**Fig. 3:**
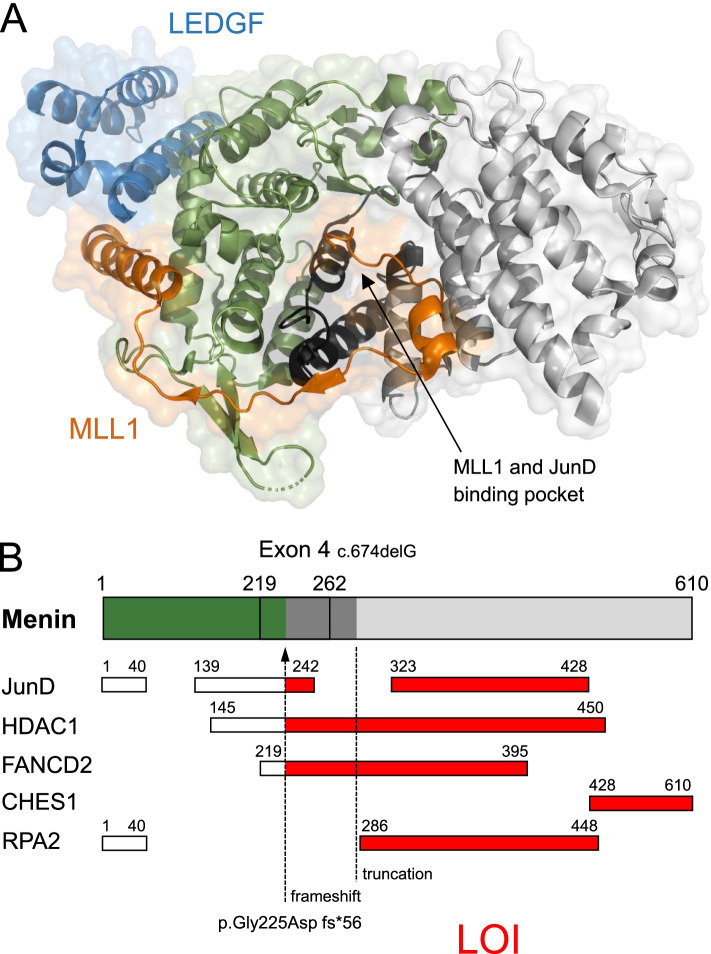
Predicted impact of the c.674delG mutation on protein-menin interactions. **A** Ternary structure of menin complexed with the mixed lineage leukaemia protein 1 (MLL1, orange) and the chromatin binding factor lens epithelium-derived growth factor (LEDGF, blue) at 3.00 Å resolution from PDB 3U88 [8]. The protein backbone is given as a cartoon model (green, dark and light gray). The region of the menin protein potentially impacted by frameshift is colored dark gray and part of the menin protein not translated colored light gray. **B** Location of the novel mutation and interaction partners of menin with predicted LOI [8, 10, 12, 13]. CHES1, checkpoint suppressor 1; FANCD2, Fanconi anemia group D2 protein; HDAC1, histone deacetylase 1; LEDGF, lens epithelium-derived growth factor; LOI, loss of interaction; MLL1, mixed lineage leukaemia protein 1; RPA2, replication protein A2

To characterize the potential impact of the p.Gly225Aspfs*56 mutation on menin interaction partners, we retrieved available structures of unbound menin and menin in complex with several interaction partners [8, 10] from the Protein Data Bank RCSB PDB [11]. The crystal structures reveal a deep common pocket for binding of short peptides of MLL1 or JunD, and another distinct protein interaction site for menin with the chromatin-anchoring protein LEDGF, formed by both menin and MLL1 [8, 10] (Fig. 3A). The topology of protein-interaction sites with menin is mapped on the menin sequence in Fig. 3B [8, 10, 12]. Based on the experimental protein structures and sequence mapping, we predicted that the p.Gly225Aspfs*56 mutation affects protein interactions of menin with multiple interacting protein partners.

**Fig. 4:**
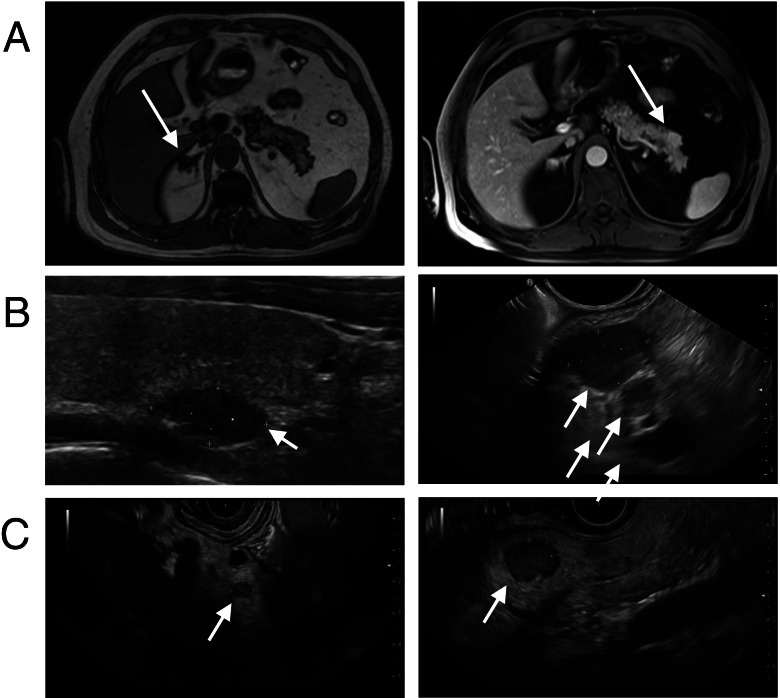
Imaging in the family members of the index patient. Imaging is showing adrenal and parathyroid adenomas and hormone-inactive NET of the pancreas. **(A)** 58-year-old father. MRI abdomen in-phase (IP) showing an adenoma of the right adrenal gland (left) and out-of-phase (OOP) MRI showing one lesion in the pancreatic tail (right). **(B)** 19-year-old brother. Ultrasound of the neck showing a right caudal parathyroid adenoma (left) and EUS with two neighboring lesions in the pancreatic tail (right). **(C)** 26-year-old sister. EUS showing multiple masses of the pancreas

**Fig. 5:**
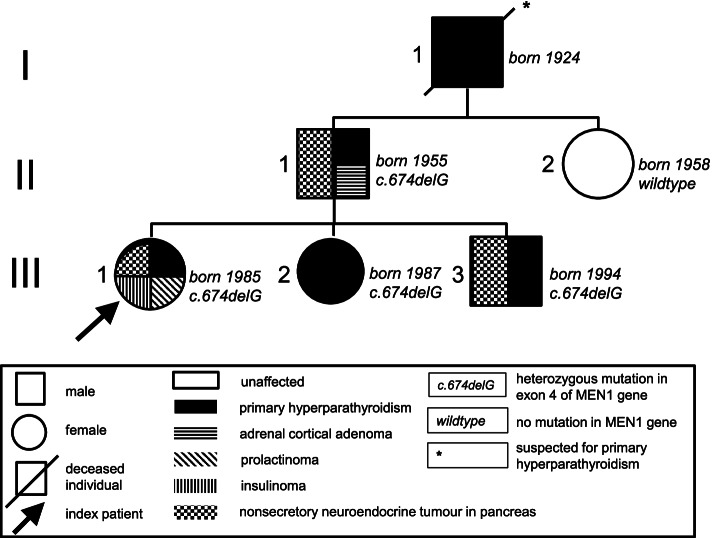
Pedigree of the MEN1 family. The family tree is shown over three generations with the index patient indicated by an arrow

In the following, we screened family members of the index patient for clinical manifestations of MEN1 and mutations in the MEN1 gene. The 58-year-old father suffered from nephrolithiasis for about 25 years, which we could correlate with primary hyperparathyroidism (Table 1). MRI showed multiple lesions in the pancreatic tail (diameter 2.1 cm) (Fig. 4A) without clinical symptoms or pathological findings in laboratory tests (Table 1). We classified the pancreatic lesions as nonsecretory tumors. Furthermore, we find a nonsecretory tumor in the right adrenal gland (Fig. 4A). We detected no adenomas of the pituitary gland. The 19-year-old brother and 26-year-old sister of the index patient were each diagnosed with primary hyperparathyroidism (Table 1). In the brother, we find nonfunctioning lesions (diameter 1.8 cm) in the pancreatic tail (Fig. 4B). In the sister, the first manifestation of MEN1 was primary hyperparathyroidism. Later, a prolactinoma treated with cabergoline and multiple masses of hormone-inactive NET were diagnosed (Fig. 4C). The 56-year-old paternal aunt of the index patient showed no hints for MEN1 in her medical history. Laboratory tests for calcium and prolactin were in the normal range (not shown). Sequencing analysis of the MEN1 gene in the father, brother and sister of the index patient detected the identical heterozygous c.674delG mutation in exon 4 than in the index patient, whereas the paternal aunt showed no corresponding MEN1 mutation. The pedigree of the MEN1 family is shown in Fig. 5.

## Discussion and conclusions

The novel mutation we describe here results in a classical MEN1 phenotype with primary hyperparathyroidism and multifocal pancreatic neuroendocrine tumors. The c.674delG; p.Gly225Aspfs*56 mutation causes a deletion at position c.674, resulting in an amino acid frameshift and a consecutive amino acid sequence change with early termination of translation of the menin protein. The protein truncation is predicted to affect sites involved in protein-menin interactions potentially contributing to the complex clinical patterns of neuroendocrine tumors and distinct clinical phenotypes in the index patient and three family members harboring p.Gly225Aspfs*56. Specifically, the p.Gly225Aspfs*56 mutation potentially causes a near complete loss of interaction (LOI) with Fanconi anemia group D2 protein (FANCD2) [13] and a complete LOI with the forkhead transcription factor checkpoint suppressor 1 (CHES1) [12], also known as FOXN3. CHES1 is a component of a transcriptional repressor complex comprising mSin3a, as well as histone deacetylase (HDAC) 1 and 2 [14]. The complex interacts with menin in an S-phase checkpoint pathway related to DNA damage response that represses the expression of multiple genes and negatively regulate protein biosynthesis [14]. Notably, aggressive tumor growth with hepatic metastasis from pancreatic neuroendocrine tumors is a major cause of death in MEN1 and observed in about 23% of patients [15]. The expression of CHES1 is reduced in many types of cancers [16]. Truncating mutations in MEN1, in particular those leading to LOI with CHES1, potentially have a higher risk of malignancy with aggressive disease courses and disease-related death, as reported earlier [12]. Other protein interactions that may contribute to higher mortality risk as a consequence of MEN1-associated neoplasia, particularly interactions with JunD [17], are at least partially conserved in p.Gly225Aspfs*56 based on in silico analysis (Fig. 3B). Although p.Gly225Aspfs*56 potentially causes complete CHES1-LOI, we did not observe aggressive disease progression over an 8-year period in the index patient and her family members with proven pancreatic neuroendocrine tumors.

In summary, we report on a novel MEN1 germline mutation causing a frameshift and early premature truncation of the menin protein with distinct clinical phenotypes and no genotype–phenotype correlation. Although predicted to cause LOI to multiple interacting proteins including CHES1, the novel mutation did not lead to aggressive pancreatic neuroendocrine tumors in long-term follow up.

## Data Availability

N/A.

## Abbreviations

CHES1: Checkpoint suppressor 1
cDNA: Complementary
DNA: Deoxyribonucleic acid
EUS: Endoscopic ultrasound
FANCD2: Fanconi anemia group D2 protein
HDAC: Histone deacetylase
LEDGF: Lens epithelium-derived growth factor
LOI: Loss of interaction
MEN: Multiple endocrine neoplasia
MLL1: Mixed lineage leukaemia protein 1
MRI: Magnetic resonance imaging
NET: Neuroendocrine tumor
NGS: Next generation sequencing
PCR: Polymerase chain reaction
PDB: Protein Data Bank
PPPD: Pylorus-preserving pancreaticoduodenectomy
PTH: Parathormone
